# Knowledge and Practice of Emergency Physicians Regarding Food-borne Disease Surveillance at Hamad General Hospital in Qatar

**DOI:** 10.7759/cureus.4934

**Published:** 2019-06-18

**Authors:** Mohamad A Chehab, Mohamed Nour, Geoffrey Bryant, Adel Zahran, Ayman Al-Dahshan, Mohamed O Bala, Noora J AlKubaisi, Nagah A Selim

**Affiliations:** 1 Preventive Medicine, Hamad Medical Corporation, Doha, QAT; 2 Preventive Medicine, Ministry of Public Health, Doha, QAT; 3 Emergency Medicine, Hamad Medical Corporation, Doha, QAT; 4 Preventive Medicine, Primary Health Care Corporation, Doha, QAT; 5 Preventive Medicine, Cairo University School of Medicine, Cairo, EGY

**Keywords:** foodborne disease, surveillance, emergency medicine, knowledge, practice, qatar

## Abstract

Introduction

According to the World Health Organization (WHO), foodborne diseases (FBD’s) have become a global health issue. In Qatar, foodborne diseases are among the top ten events reported to the Ministry of Public Health. Efforts to enhance FBD surveillance cannot succeed without involving the emergency department (ED), which is typically the first point of contact for the FBD victims with the healthcare system. Therefore, we aimed to explore the knowledge and practices of emergency physicians regarding stool sample collection as part of FBD surveillance efforts in Qatar.

Methods

A cross-sectional study was conducted at the ED of Hamad General Hospital (HGH) between July 22 and September 12 of 2018. The enrolled participants were invited to participate in an online survey at the “QSurvey” platform. The data was analyzed using Microsoft Excel (Version 2016). Descriptive statistics such as frequency tables, proportions, and percentages were applied as appropriate.

Results

A total of 65 responses (response rate: 29.27%) were received within the duration of the study. Most participants were specialists (45%), graduated between 2000 and 2013 (64%), and worked for one year or more at HGH-Hamad Medical Corporation (95%). Regarding their knowledge of FBD surveillance, most participants (80%) reported that a stool culture is a necessary laboratory investigation for patients with acute bloody diarrhea and fever. Also, a large percentage of physicians identified salmonella (75%), Clostridium difficile (70%), and E.coli O157:H7 (70%) as pathogens of nationally notifiable diseases. Regarding the respondents’ practice towards FBD surveillance, almost three-quarters of the physicians (72%) who encountered a patient with acute diarrhea did not order a stool culture. Subsequently, about two-thirds (62%) of the participants who requested a stool culture reported not following up on the results of such request. Regarding the history taken from patients with acute diarrhea, a large percentage of respondents reported asking about the patient’s travel history (100%), presence of any sick contacts (93.6%), and presence of any associated symptoms (abdominal pain, fever, bloody stool) as well as other details.

Conclusion

The current research identified several gaps regarding the knowledge and practice of emergency physicians towards the surveillance of foodborne disease. Such results serve as a basis for future research and intervention strategies to augment surveillance activities related to food-borne diseases in the State of Qatar.

## Introduction

According to the World Health Organization (WHO), foodborne diseases (FBD) include a wide spectrum of illnesses and have become a global health issue. FBD’s result from the consumption of foods contaminated with microorganisms, their toxins, or chemicals [1]. The most common symptom of foodborne disease is acute diarrhea, but the clinical picture might deteriorate in some cases and cause kidney and liver disease, neurologic disorders, and even death. The WHO’s first-ever report of the global burden of foodborne diseases in 2015, revealed that these illnesses affect 600 million people annually, with a staggering 420,000 deaths and 25 million DALY’s [2]. Furthermore, the Centers for Disease Control and Prevention (CDC) reported that foodborne diseases were responsible for 3,000 deaths and 128,000 hospitalizations in the United States of America during 2011 [3]. Moreover, many of the bacteria behind countless food poisoning outbreaks globally (salmonella, E.coli, Shigella, and Staphylococcus aureus) have been prioritized by the WHO as antibiotic-resistant organisms that necessitate developing novel antimicrobials [4]. 

The Eastern Mediterranean Region (EMR) ranks third on the global scale in regards to the estimated burden of FBD’s per population, following the African and South-East Asian Regions. In addition to that, it is estimated that the health authorities in the EMR report more than 100 million cases (101,699,626) of FBD annually; a third of which are children under five years of age. Also, the region reports a median of 6 FBD-related deaths per 100,000 totaling at 36,525 FBD-related deaths annually [2]. 

Efforts to enhance FBD surveillance cannot succeed without involving the emergency department (ED), which is typically the first point of contact for the FBD victims with the healthcare system. Effective interventions need to be based on a comprehensive understanding of the level of knowledge and practice of the ED staff. A serious FBD outbreak can easily go undetected or be discovered late due to the lack of appropriate knowledge on FBDs or lack of awareness of the available notification channels among several other factors. An earlier study among American emergency physicians revealed that over one-third of them (38%) ordered stool cultures for patients with acute diarrhea, half of them knew about two selected notifiable diarrhea-causing pathogens, and a minority were familiar with the clinical history taking that is of public health significance [5]. ED physicians can play a pivotal role in raising awareness among their patients about FBD’s as well as relevant preventive strategies. 

While different healthcare providers share the responsibility of delivering healthcare services in Qatar, the Health Protection and Communicable Diseases department (HP & CDC) at the Ministry of Public Health (MoPH), bears the sole responsibility of investigating and responding to epidemics such as FBD outbreaks. In addition, foodborne diseases are among the top ten events reported to the HP and CDC department. Therefore, Qatar’s Public Health Strategy (2017-2022) identified FBDs as one of the priority areas where the action will include augmenting surveillance and control capacities, starting with the ED. However, the capacity building process should be based on a sufficient understanding of the baseline situation at the emergency department and the knowledge and practices of its staff in relation to FBD. Moreover, Hamad Medical Corporation is the major governmental provider (90%) of acute health services in the country, receiving a total of 1,119,951 emergency department visits across its various healthcare facilities [6]. Given the scarcity of information on the subject, we decided to target ED physicians with a survey to explore their knowledge and practices regarding stool sample collection as part of FBD surveillance efforts in Qatar.

## Materials and methods

Study design

This was a descriptive cross-sectional study conducted at the ED of Hamad Medical Corporation’s Hamad General Hospital (HMC-HGH), which constitutes the main provider of the secondary and tertiary health services in Qatar. It has been approved by the Medical Research Center (MRC-HMC) as a quality improvement project under protocol number MRC-01-17-110.

Study population

The study targeted all emergency physicians, of different training or specialty levels, at the ED of HGH. However, physicians engaged in Infection Prevention and Control activities were excluded from being invited to the current study.

Sampling technique

A convenient sampling technique was employed and the targeted ED physicians were invited through email or the departmental WhatsApp group. Those who opted to participate were asked to complete an online survey based in the “QSurvey” platform between July 22 and September 12 of 2018. Invitations were followed by reminders until the calculated sample size was satisfied.

Sample size

The required sample size was 49 respondents based on the sample size calculation formula for cross-sectional studies [7], where Z is the standard normal variate (at 5% type I error, it is 1.96), p is the expected proportion of notifiable FBD knowledge in the population based on an earlier study (50% or 0.5) [5], and d is the absolute error or precision (10% or 0.1).

Outcome measures

The primary outcome variables were the knowledge and practice of emergency physicians towards FBD surveillance. To assess the physicians’ knowledge of FBD outbreak surveillance, a list of notifiable FBDs was obtained from the HP and CDC department at the MoPH. The practices of the potential participants were assessed through questions regarding their requesting of stool analysis for patients presenting with acute diarrhea. 

Questionnaire

After reviewing relevant data collection tools from the literature (as those utilizing the Hennessy-Hicks Training Needs Analysis questionnaire) [8] and obtaining a list of notifiable FBD's from the HP and CDC department at the MoPH, an anonymous, self-administered, and electronic questionnaire was developed. The questionnaire was formulated in the English language and was comprised of four sections: the employment history of the respondent (three questions), the knowledge of FBD surveillance (three questions) and the practice (five questions). The eleven questions of the survey varied between close-ended, dichotomous, and multiple choice questions.

The questionnaire’s face validity was achieved through consulting four experts in the field from different health institutions across the country. Before launching the study, the questionnaire was piloted among a small sample of the study population and subsequent modifications were made to make the questionnaire more reader-friendly and comprehensible.

Data analysis plan

The obtained data were analyzed using Microsoft Excel (Version 2016). Descriptive statistics such as frequency tables, proportions, and percentages were applied as adequate. Chi-square test of significance was employed for comparison between categorical variables.

## Results

Survey responses were received from a total of 65 emergency physicians at HGH within the duration of the study. Less than half of the respondents reported being specialists (45%) while the rest were dispersed between fellows (26%), consultants (15%), emergency medicine residents (12%), and rotating residents (2%). Regarding their year of graduation, almost two-thirds (64%) of participants graduated between 2000 and 2013, with the earliest graduate was from 1983 and the latest one being in 2017. Also, the vast majority of participating physicians (95%) reported working for one year or more at HGH-HMC, while the rest (5%) reported working at the institution for less than a year. Further stratification of the aforementioned variable revealed that more than half (55%) of the physicians in this survey were employed at HMC from 1-5 years, while more than one-third (37%) worked between 6 and 10 years.

**Figure 1:**
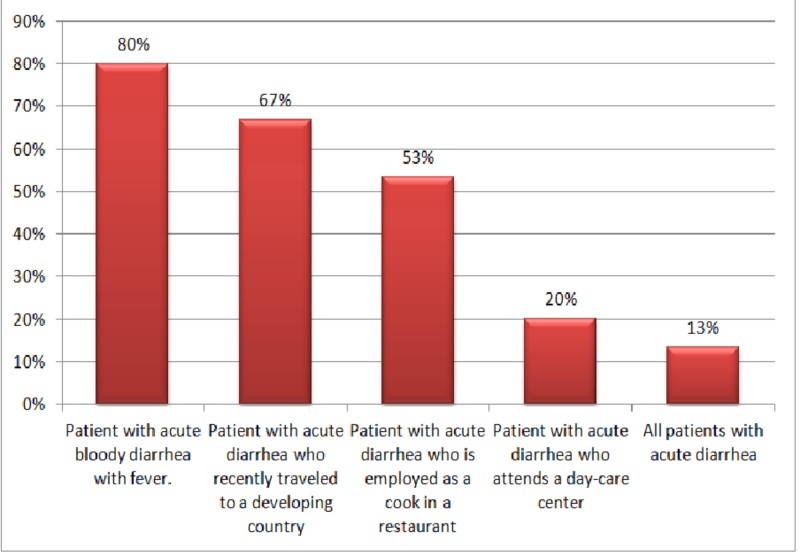
Distribution of physicians' responses regarding the indications for a stool culture

Regarding their knowledge, more than three-quarters of respondents (78%) acknowledged the presence of a clinical guideline for the management of infectious diarrhea at HMC. When asked about their knowledge on the management of infectious diarrhea at HMC, most participants (80%) reported that a stool culture is recommended as a laboratory investigation for patients presenting with acute bloody diarrhea and fever (Figure 1).

**Figure 2:**
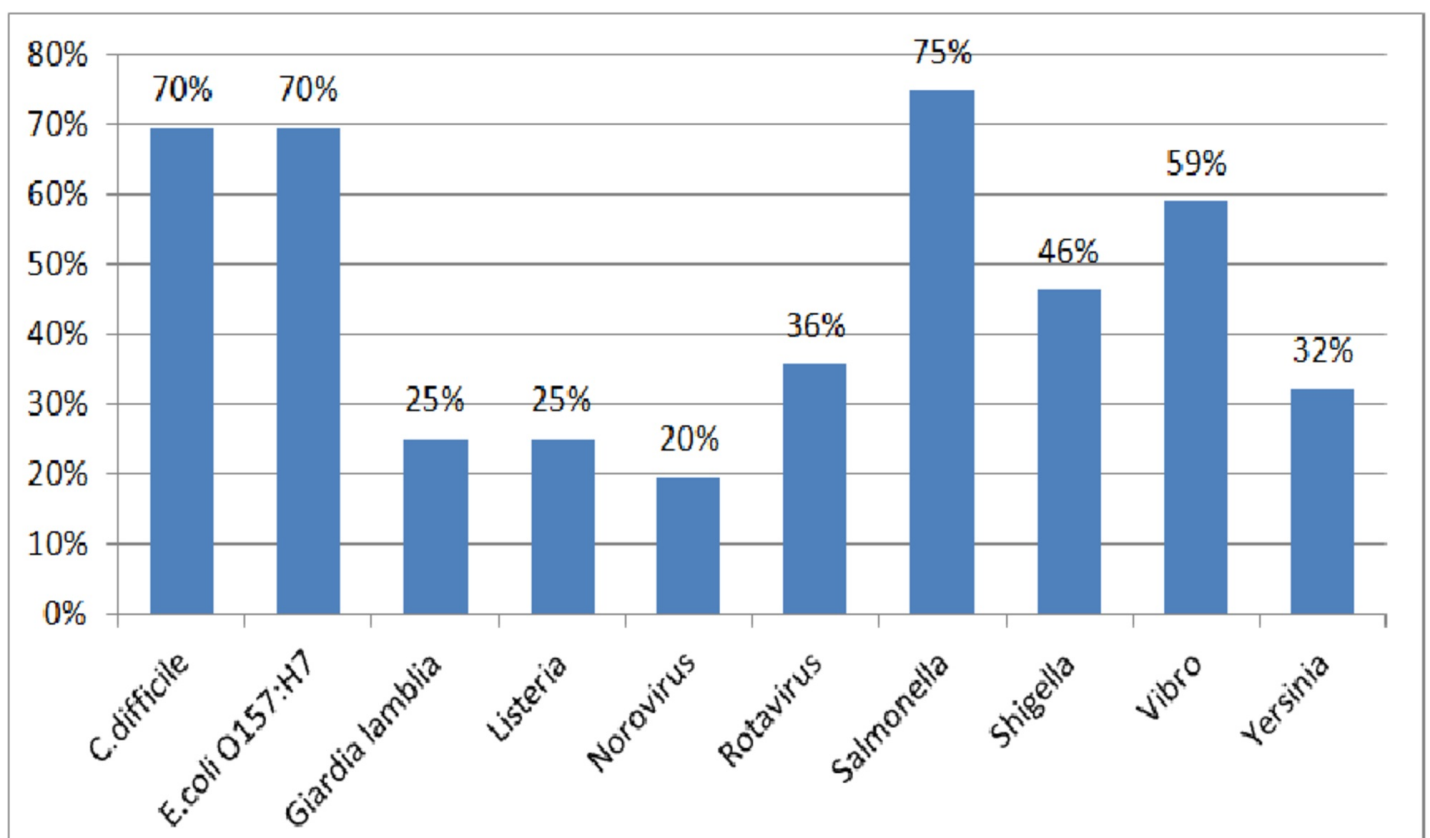
Distribution of participants' responses regarding pathogens notifiable to the Ministry of Public Health

Furthermore, a large percentage of physicians identified Salmonella (75%), Clostridium difficile (70%), and E.coli O157:H7 (70%) as pathogens of nationally notifiable diseases. On the other hand, norovirus was the least reported pathogen to be notifiable to the national health authority (Figure 2).

Regarding their practice, the majority of participants (84%, n=47) reported encountering a patient with acute diarrheal illness in the Emergency Department during the past month. Among those, less than a quarter (23%) examined such patients earlier in the month (8-30 days), almost half (47%) saw patients with acute diarrhea within the past week (2-7 days), and almost a third (30%) during the earlier day (≤1 day). Subsequently, most of the aforementioned physicians (72%, n=34) declared not ordering a stool culture for patients with acute diarrhea. Among the respondents who requested a stool culture, about two-thirds (62%, n=8) reported not following up on the results of their request. Regarding the history taken from patients with acute diarrhea, a large percentage of respondents reported asking about the patient’s travel history (100%), presence of any sick contacts (93.6%), and presence of any associated symptoms (abdominal pain, fever, bloody stool) as well as other details (Table 1).

**Table 1:** Distribution of responses regarding physicians' history-taking practices

Answer Choices	Responses
Did you travel in the week prior to your illness?	100.0%
Have you been experiencing abdominal pain with diarrhea?	97.9%
Have you noticed blood in your stool?	97.9%
Have you been experiencing fever with diarrhea?	95.7%
Do you know anyone else ill with similar symptoms around the same time as you?	93.6%
How long have you had diarrhea?	93.6%
What is your occupation?	68.1%
Do any of your close contacts work in occupations that involve contacts with patients or children?	61.7%

Chi-square (Fisher's exact test) analysis revealed no significant difference regarding the practice of stool collection among physicians of different training levels (F=0.077, p=0.782). Similarly, there was no statistically significant difference (F=2.923, p=0.939) regarding the practice of stool collection among physicians with different years of experience.

Several factors affected the respondents’ willingness to order a stool culture for patients with acute diarrhea, such as the likelihood that the culture’s result will alter the patient’s management (66%), the likelihood that the culture will identify a specific pathogen (50%), the time needed for a stool culture to yield results (48%), the time needed by a patient to submit a stool specimen (28.6%), and the patient’s willingness to undergo a stool culture (26.8%).

Finally, when asked about which single system-based change would enable them to order a stool culture for a patient with acute diarrheal illness, more than half of the participating physicians (57.1%) supported the automatic notification of the stool culture result to the communicable disease control section- MoPH. Also, less than third of the participating physicians (30.4%) preferred the development of an easier system of stool sample delivery by the patient to the designated authority at HMC and the Primary Health Care Corporation health centers (Table 2).

**Table 2:** Distribution of physicians' responses on system-based changes for amplifying FBD surveillance in the ED FBD: foodborne diseases; ED: emergency department; MoPH: Ministry of Public Health; HMC: Hamad Medical Corporation; PHCC: Primary Health Care Corporation.

Answer Choices	Responses
Automatic notification of the stool culture result to the communicable disease control section- MoPH	58.2%
Easier system of stool sample delivery by the patient to the designated authority (HMC, PHCC)	30.9%
If the emergency physician was not obligated to follow up on the result of the stool culture	7.3%

## Discussion

This was a descriptive cross-sectional study that sought to explore the knowledge and practices of physicians at the emergency department of the largest secondary care provider in Qatar with regard to the surveillance of FBDs, particularly requesting stool culture and reporting acute diarrhea to MoPH.

The study revealed that a significant proportion of the respondents is not adequately aware of the notifiable pathogens related to foodborne diseases. The majority (75%) rightfully selected salmonella while a similar proportion (70%) wrongly believed that Clostridium difficile is among the designated pathogens required for reporting to MoPH. Similarly, a cross-sectional study among hospital physicians in Spain showed that the majority (70.5%) were unable to identify the full list of notifiable diseases in the country. Such lack of knowledge reflected poorly on the physicians’ notification practices and was associated with underreporting [9].

Despite the fact that most participants (80%) in the current study reported stool culture to be a necessary laboratory investigation for patients with acute bloody diarrhea and fever, only a few (28%) ordered a stool culture when encountering a relevant patient in the ED. On the other hand, an earlier study conducted in the US reported a higher figure (38%) of requesting stool culture for diarrheal patients attending the emergency department [7]. Given the fact that emergency physicians operate in an acute stressful setting with a high turnover of patients, the ability to provide care for a specific patient over a long duration of time can be challenging because stool cultures might require days to yield results.

In the present study, the ED physicians acknowledged seeing patients with acute diarrhea on a daily, weekly, or monthly basis. However, a patient with acute diarrhea might not rank first on the prioritization list of an emergency physician. This was evident in the study when two-thirds (62%) of the participants who requested a stool culture did not follow up on the results of such a request. Optimally, stool analysis in the ED should serve as a guide for physicians on subsequent management protocols and if necessary the involvement of public health officials for outbreak control [10].

Among the factors that influenced the respondents’ willingness to order a stool culture for patients with acute diarrhea, the likelihood that the culture’s result will alter the patient’s management (66%) and identify a specific pathogen (50%) as well as the time needed for a stool culture to yield results (48%) were the three most commonly reported ones. In addition, the ED physicians in the current study were keen to elicit epidemiological evidence such as occupation, travel history, and contact with sick people. The aforementioned findings align well with the potential public health role of emergency medicine as a specialty. However, the discipline requires adequate resources, incentives, and organizational changes to fulfill that role [11]. For example, a strategic partnership between the CDC department at the MoPH and the ED at HGH might prove beneficial in augmenting FBD surveillance efforts in the country. Such a partnership will result in a mutual understanding of the FBD burden in Qatar, engagement of the emergency medicine physicians in FBD surveillance, capacity building, and research opportunities.

Nevertheless, nearly half of the participants expressed their lack of enthusiasm to request stool cultures if the time, until a result was obtained, was relatively long or if the healthcare facility was not appropriately equipped. Similarly, emergency physicians were unlikely to request stool culture from seemingly unwilling patients. In addition, a focus group of healthcare professionals in the United States of America revealed that the common themes for not ordering a stool culture when needed were variable and included the lack of information among physicians as well as patients, embarrassment or non-cooperation of the patients, and challenges related to the stool sample collection process [12]. Another qualitative study among patients in the United Kingdom found that some of the reported barriers were the feeling of discomfit, hygiene-related concerns, privacy-related issues, lack of knowledge, and fear of the results [13].

The participating physicians’ duration of work at HMC did not have a substantial effect on their practice towards reporting FBDs and requesting laboratory evidence. However, more than half of the respondents (57.1%) supported the automatic notification of the stool culture results to the communicable disease control section at MoPH as a systematic solution to the reporting of foodborne diseases. Moreover, the vast majority of physicians (91%) agreed that an online notification option, based in the current electronic medical record system, would facilitate their reporting of foodborne diseases to the health authorities. On the other hand, a systematic review of the timeliness of infectious disease notification systems revealed that although automatic or electronic systems were faster than the conventional ones, the former still required significant operational efforts to satisfy the predefined notification timeframes [14].

The current study has several strengths and limitations. First of all, the study was the first of its kind to evaluate the knowledge and practices of ED physicians towards FBD surveillance efforts in Qatar. Also, the calculated sample size was fulfilled and the study results can be generalized to other ED physicians working in the same setting. In addition, the use of a validated questionnaire to collect data through an online platform added scientific merit to the study results. Nevertheless, the current study is not without limitations. The self-administering format of the questionnaire may have introduced self-reporting bias into the study. In addition, the response among the target population might have been associated with volunteer bias. Finally, the knowledge of the participants on FBD surveillance was not quantified in this research. Thus, it was not possible to evaluate any association between the physicians’ characteristics and their knowledge.

## Conclusions

In conclusion, the current research identified several gaps regarding the knowledge and practices of emergency medicine physicians towards the surveillance of foodborne disease. The yielded results serve as a basis for future research and intervention strategies to augment surveillance activities related to food-borne diseases in the State of Qatar. The outcomes also highlight the need for updated as well as nationally-relevant clinical protocols and guidelines to improve service delivery across different health care institutions in the country.
